# Terahertz large-area unidirectional surface magnetoplasmon and its applications

**DOI:** 10.1038/s41598-023-49348-y

**Published:** 2023-12-11

**Authors:** Qian Shen, Jinhua Yan, Yun You, Senpeng Li, Linfang Shen

**Affiliations:** 1 College of Science, Zhejiang University of Technology, Hangzhou, 310023 China; 2 College of Information Engineering, Zhejiang University of Technology, Hangzhou, 310023 China

**Keywords:** Magneto-optics, Nanophotonics and plasmonics

## Abstract

Unidirectional surface waves in nonreciprocal plasmonic platforms with nonlocal effects have been a topic of significant interest and some controversy. In this study, we present a scheme to achieve unidirectional surface magnetoplasmons (USMPs) with large modal areas at terahertz frequencies. Such large-area USMPs (LUSMPs) exist in a metal-UENZ (uniaxial-$\varepsilon$-near-zero)-Si-InSb structure under external magnetic field, where the effect of nonlocality is included. The field of the LUSMP extends almost uniformly in the UENZ layer with a thickness of wavelength scale, thus its modal size can be represented by the UENZ-layer thickness. Due to the modal energy primarily distributed in the thick UENZ layer, the nonlocality-induced leakage of the LUSMP is significantly reduced by an order of magnitude, compared to previous USMP existing at interface between InSb and opaque isotropic medium. Due to their large modal sizes, such LUSMPs can be efficiently excited by terahertz radiations directly from free space. In addition, LUSMPs offer high degree of freedom for manipulating terahertz waves, such as energy squeezing and trapping. Based on LUSMPs, a terahertz free-space isolator is also developed. Our findings have important implications to the development of innovative plasmonic devices in terahertz regime.

## Introduction

Unidirectional propagation of surface magnetoplasmons (SMPs), as a striking phenomenon in nonreciprocal and topological electromagnetics, have attracted growing interest and also caused some controversy. SMPs are nonreciprocal surface plasmon polaritons in plasmonic materials magnetized by external magnetic field (which breaks time-reversal symmetry), and their studies began five decades ago^1–4^. In recent years, SMPs have received a resurgence of interest due to possibility of realizing truly unidirectional transport channels^5–21^. For nonreciprocal plasmonic platforms, there may exist unidirectional frequency windows, in which SMPs are allowed to propagate only in one direction. Such unidirectional SMPs (USMPs) can even be immune to backscattering at imperfections and defects. Remarkably, when one-way channel is terminated, USMPs might be completely trapped, resulting in an accumulation of electromagnetic energy with high field localization^16–18^. Thus, an apparent violation of the time-bandwidth limit is achieved^19^. However, subsequent studies argued that USMPs reported in Ref.^19^ are not strictly unidirectional when nonlocal material effects are considered^20^. Recently, true USMPs that are robust to nonlocal effects have been reported^21^. Such USMPs exist at the interface between magnetized plasma and opaque medium and are topologically protected. However, due to the nonlocality, these USMPs become leaky and suffer from serious leakage losses^21^. For a typical example in Ref.^21^, USMP only has a propagation length shorter than two (vacuum) wavelengths even in the absence of material loss, and this would hinder its practical applications. Hence, it is necessary to develop alternative types of USMPs that can completely eliminate or significantly mitigate the impact of nonlocal effects.

As surface waves, the fields of USMPs are generally confined in a narrow boundary. Recently, unidirectional modes with large modal areas have been reported. In the microwave regime, researchers have demonstrated large-area unidirectional modes in pseudospin-field-dependent waveguides and photonic crystals containing Dirac points^22,23^. Large-area USMPs (LUSMPs) have also been achieved in the microwave regime by using a uniaxial $\mu$-near-zero material^24^. For such LUSMPs, most of the modal energy is distributed within the thick $\mu$-near-zero material layer. Therefore, it is naturally desired that LUSMP mode can be extended to terahertz (THz) regime. In recent years, rapid development in terahertz science and technology has been made because of huge potential in imaging, spectroscopy, biomedical sciences, and communications. For those applications, terahertz USMPs have been proposed^7,21^, which are of particular importance for functional devices such as isolators, switches and splitters. Such USMPs can be achieved using semiconductors under external magnetic field, and they may be classified into two types. The first-type USMPs are the conventional SMPs, and exist in the lower bandgap of the magnetized semiconductor^7^, but they have been recently shown to lose their strict unidirectional nature if nonlocal effects are included^20^. The second-type USMPs are true unidirectional modes, existing in the upper bandgap of the magnetized semiconductor, and they are robust to nonlocality because the upper bandgap is topologically nontrivial^21^. However, these robust USMPs still suffer from serious leakage caused by the nonlocality. If LUSMPs could be achieved at terahertz frequencies, the impact of nonlocal effects in plasmonic waveguides would be largely weakened and the modal leakage can be reduced to a very low level. In addition, LUSMPs can also provide high degree of freedom to manipulate terahertz waves.

**Figure 1:**
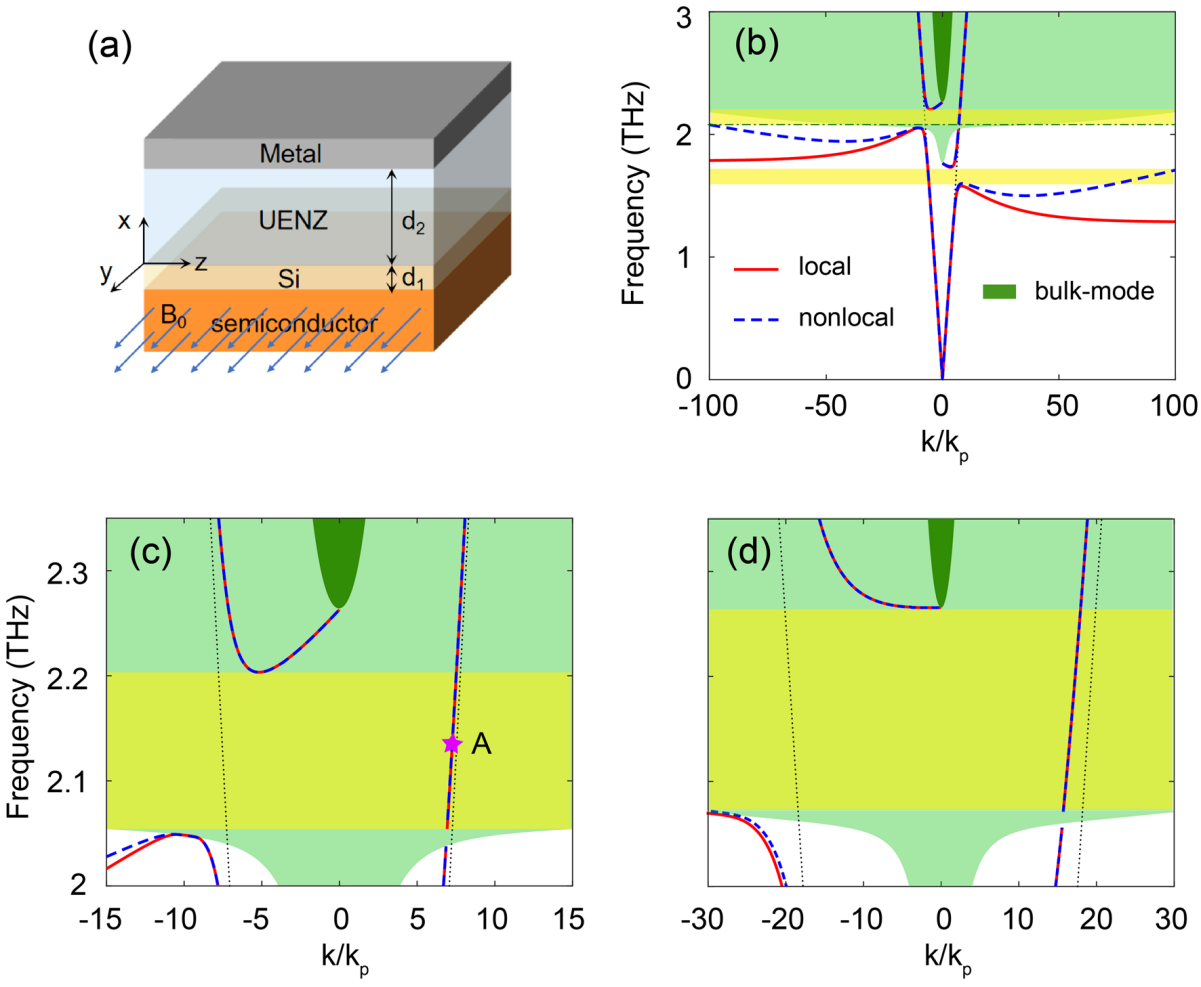
(**a**) Schematic of the metal-UENZ-Si-InSb waveguide. (**b**) Dispersion diagram for the waveguide. Solid and dashed lines represent the dispersion curves for SMPs in the local ($\beta = 0$) and nonlocal ($\beta = 1.07 \times 10^6$ m/s) models, respectively. The dotted lines are light lines in the UENZ material, and the green shaded areas represent the bulk-mode zones in the magnetized InSb for the nonlocal model. But the lower limit of the upper bulk-mode bandgap for the local model is indicated by the dot-dashed line. In the nonlocal model, the lower bulk-mode zone covers the upper bandgap, thus it becomes an incomplete bandgap. Moreover, USMP vanishes in the lower bandgap. The parameters of the Si and UENZ layers are $\varepsilon _z=0.001$, $\varepsilon _x=50$, $d_1=0.63$ $\upmu$m, and $d_2=300$ $\upmu$m, and the plasma frequency of the InSb is $f_p=\omega _p/2\pi =2$ THz. (**c**) Magnified view of the USMP window in (**b**). The unidirectional window in (**c**) ranges from 2.06 THz to 2.2 THz. (**d**) Dispersion curves for SMPs in the local (solid) and nonlocal (dashed) models, where $\varepsilon _x=300$ and $d_1=0.18$ $\upmu$m, and the other parameters are the same as in (**b**) and (**c**). In this case, the whole upper bandgap is a unidirectional window.

In this paper, we propose a scheme to achieve terahertz LUSMPs using uniaxial $\varepsilon$-near-zero (UENZ) material^25^. Different from all previous terahertz USMPs, which are tightly localized on the semiconductor surface, the proposed terahertz LUSMPs can possess modal spots with sizes larger than a wavelength. Such terahertz LUSMPs exist in the upper bandgap of the magnetized semiconductor, so they are topologically protected and robust to nonlocality. More importantly, compared to previous (true) terahertz USMPs, the nonlocality-induced leakages of LUSMPs at terahertz frequencies can be significantly reduced by an order of magnitude, because their modal energies are mainly distributed in the thick UENZ layer. Owing to their large modal spots, these LUSMPs can be efficiently excited by terahertz radiation incident from free space. Moreover, based on LUSMPs, we will numerically demonstrate several interesting examples of flexibly manipulating terahertz radiations.

## Physical model and dispersion diagram

The proposed waveguide for supporting terahertz LUSMP is a metal-UENZ-Si-semiconductor stratified structure under an external dc magnetic field $B_0$, as illustrated in Fig. 1a. We start with analysis of the dispersion for the waveguide in local material model. In this waveguide, the metal is assumed to be perfect electric conductor, which is a valid approximation in terahertz regime. The Si layer with thickness $d_1$ has the relative permittivity $\varepsilon _d=11.68$^20^, and the UENZ material layer with thickness $d_2$ has the relative permittivity in the form

1 $$\begin{aligned} {\mathop \varepsilon \limits ^ \leftrightarrow } _e = \left[ {\begin{array}{*{20}{c}} \varepsilon _x &{} 0 &{} 0\\ 0 &{} \varepsilon _y&{} 0\\ 0 &{} 0 &{} \varepsilon _z \end{array}} \right] , \end{aligned}$$

where $\varepsilon _x$ is larger than 1, and $\varepsilon _y=\varepsilon _z$ is close to zero. The semiconductor with gyroelectric anisotropy induced by the external magnetic field is characterized by a relative permittivity tensor

2 $$\begin{aligned} {\mathop \varepsilon \limits ^ \leftrightarrow } _s = \varepsilon _{\infty } \left[ {\begin{array}{*{20}{c}} \varepsilon _1 &{} 0 &{} i\varepsilon _2\\ 0&{} \varepsilon _3 &{} 0\\ -i\varepsilon _2 &{} 0 &{} \varepsilon _1 \end{array}} \right] , \end{aligned}$$

with

$$\begin{aligned} \begin{array}{l} {\varepsilon _{1}=1-\frac{\left( \omega +i\nu \right) \omega _{p}^{2} }{\omega \left[ \left( \omega +i\nu \right) ^{2} -\omega _{c}^{2} \right] }},\\ {\varepsilon _{2}=\frac{\omega _{c} \omega _{p}^{2} }{\omega \left[ \left( \omega +i\nu \right) ^{2} -\omega _{c}^{2} \right] }},\\ {\varepsilon _{3}=1-\frac{\omega _{p}^{2} }{\omega \left( \omega +i\nu \right) }}, \end{array} \end{aligned}$$

where $\omega$ is angular frequency, $\omega _p$ is the plasma frequency, $\nu$ is the electron scattering frequency, $\omega _{c} =eB_{0} /m^{*}$ (where *e* and $m^{*}$ are, respectively, the charge and effective mass of the electron) being the electron cyclotron frequency, and $\varepsilon _{\infty }$ is the high-frequency permittivity. The magnetized semiconductor exhibits two bandgaps for bulk modes with transverse-magnetic (TM) polarization: a lower bandgap below the plasma frequency, which exists even in the absence of external magnetic field, and an upper bandgap above the plasma frequency, which is opened by the external magnetic field. The upper bandgap has nontrivial topological property characterized by a nonzero gap Chern number, and as a result, USMP within it is topologically protected^21^ and thus could be robust to nonlocal effects.

**Figure 2:**
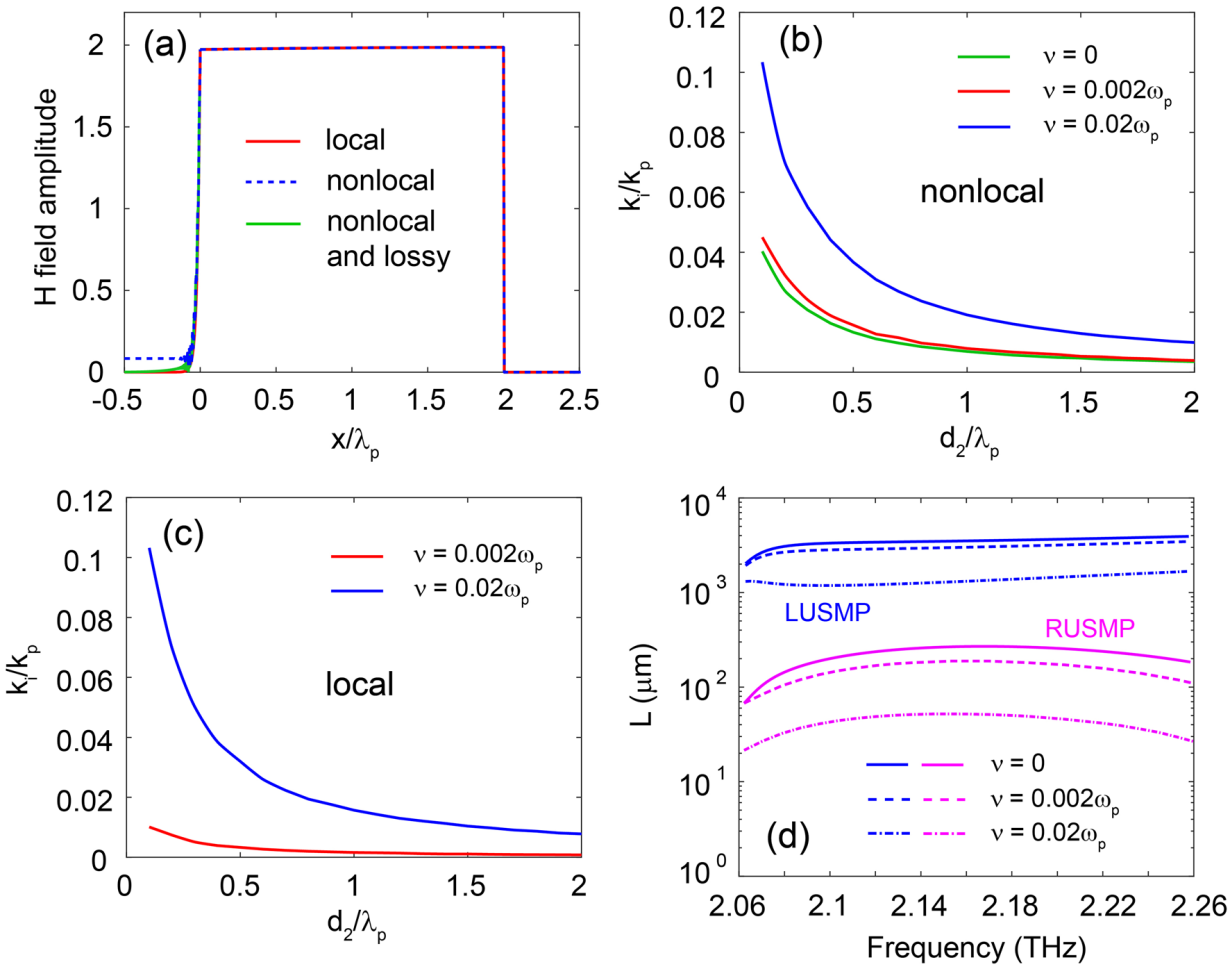
(**a**) H-field profile of LUSMP for $d_2=300$ $\upmu$m. As a leaky mode, the H-field amplitude of USMP in the nonlocal and lossless case does not decay to zero in the InSb layer. (**b**, **c**) Dependence of $k_i$ on $d_2$ in the nonlocal ($\beta = 1.07 \times 10^6$ m/s) and local ($\beta =0$) models for various $\nu$ values. The frequency is all 2.12 THz in (**a**), (**b**) and (**c**). (**d**) Propagation lengths ($L=1/2k_i$) of LUSMP and RUSMP as functions of frequency. Various $\nu$ values are considered. For the LUSMP waveguide, the UENZ layer has $d_2=300$ $\upmu$m. The other parameters are $\varepsilon _z=0.001$, $\varepsilon _x=50$, and $d_1=0.63$ $\upmu$m for all the cases.

The waveguide supports SMPs with TM-polarized field (i.e., $H=\hat{y}H_{y}$). The dispersion relation of SMPs in the local material model can be derived analytically from Maxwell’s equations combined with proper boundary conditions, and we obtain

3 $$\begin{aligned} \alpha _s + \frac{\varepsilon _2}{\varepsilon _1} k + {\varepsilon _{\mathrm{{v}}}}\frac{(\alpha _e/\varepsilon _z)\textrm{tanh}(\alpha _e d_2)+(\alpha _d/\varepsilon _d)\textrm{tanh}(\alpha _d d_1)}{(\alpha _e \varepsilon _d /\varepsilon _z \alpha _d ) \textrm{tanh}(\alpha _d d_1) \textrm{tanh}(\alpha _e d_2)+1}=0, \end{aligned}$$

where *k* is the propagation constant, $\alpha _e=\sqrt{\varepsilon _{z}\left( k^{2}/\varepsilon _{x}-k_{0}^{2} \right) }$, $\alpha _d=\sqrt{ k^{2}-\varepsilon _d k_{0}^{2}}$ ($k_0=\omega /c$ is the vacuum wavenumber), and $\alpha _s=\sqrt{ k^{2}-\varepsilon _{\mathrm{{v}}} k_{0}^{2}}$ (where $\varepsilon _{\mathrm{{v}}}=\varepsilon _{1}-\varepsilon _{2}^{2}/\varepsilon _1$) are, respectively, the transverse attenuation coefficients of the field in the UENZ material, silicon, and semiconductor. From Eq. (3), two asymptotic frequencies can be obtained, at which $k\rightarrow \pm \infty$, and they are given by

4 $$\begin{aligned} \begin{array}{l} {\omega _{sp}^{+}=\frac{1}{2} \left( \sqrt{\omega _{c}^{2}+4\omega _{p}^{2} \frac{\varepsilon _{\infty }}{\varepsilon _{\infty }+\varepsilon _d}} -\omega _{c} \right) },\\ {\omega _{sp}^{-}=\frac{1}{2}\left( \sqrt{\omega _{c}^{2}+4\omega _{p}^{2} \frac{\varepsilon _{\infty }}{\varepsilon _{\infty }+\varepsilon _d}} +\omega _{c} \right) }. \end{array} \end{aligned}$$

**Figure 3:**
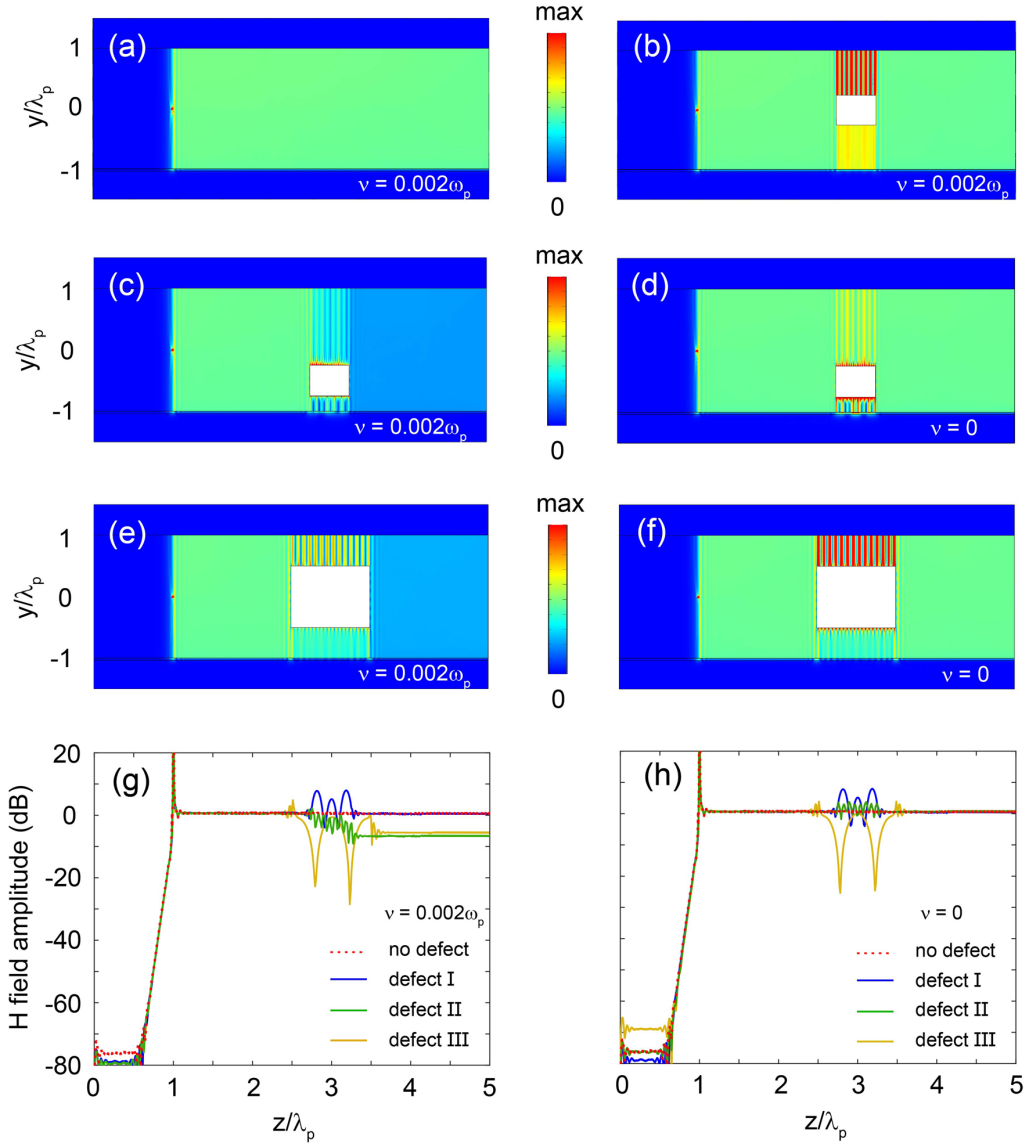
Simulations for waveguides in the nonlocal model. (**a**–**f**) Simulated magnetic field amplitudes launched by a magnetic current line source at $f = 2.12$ THz for different cases. (**a**) Lossy case without defect, (**b**) lossy case with defect I, which is a square air column with side length 75 $\upmu$m, centered in the middle of the UENZ layer. Note that the field patterns for the lossless cases without defect or with defect I are almost identical to those in (**a**) and (**b**). (**c**, **d**) Lossy and lossless cases with defect II, which is the defect I shifted down by 75 $\upmu$m. (**e**, **f**) Lossy and lossless cases with defect III, which is the doubled defect I. (**g**, **h**) Distributions of magnetic field amplitude along the middle line of the UENZ layer for the lossless and lossy cases without or with defects. For all the lossy cases, $\nu =0.002\omega _p$. The other parameters are the same as in Fig. 1b.

**Figure 4:**
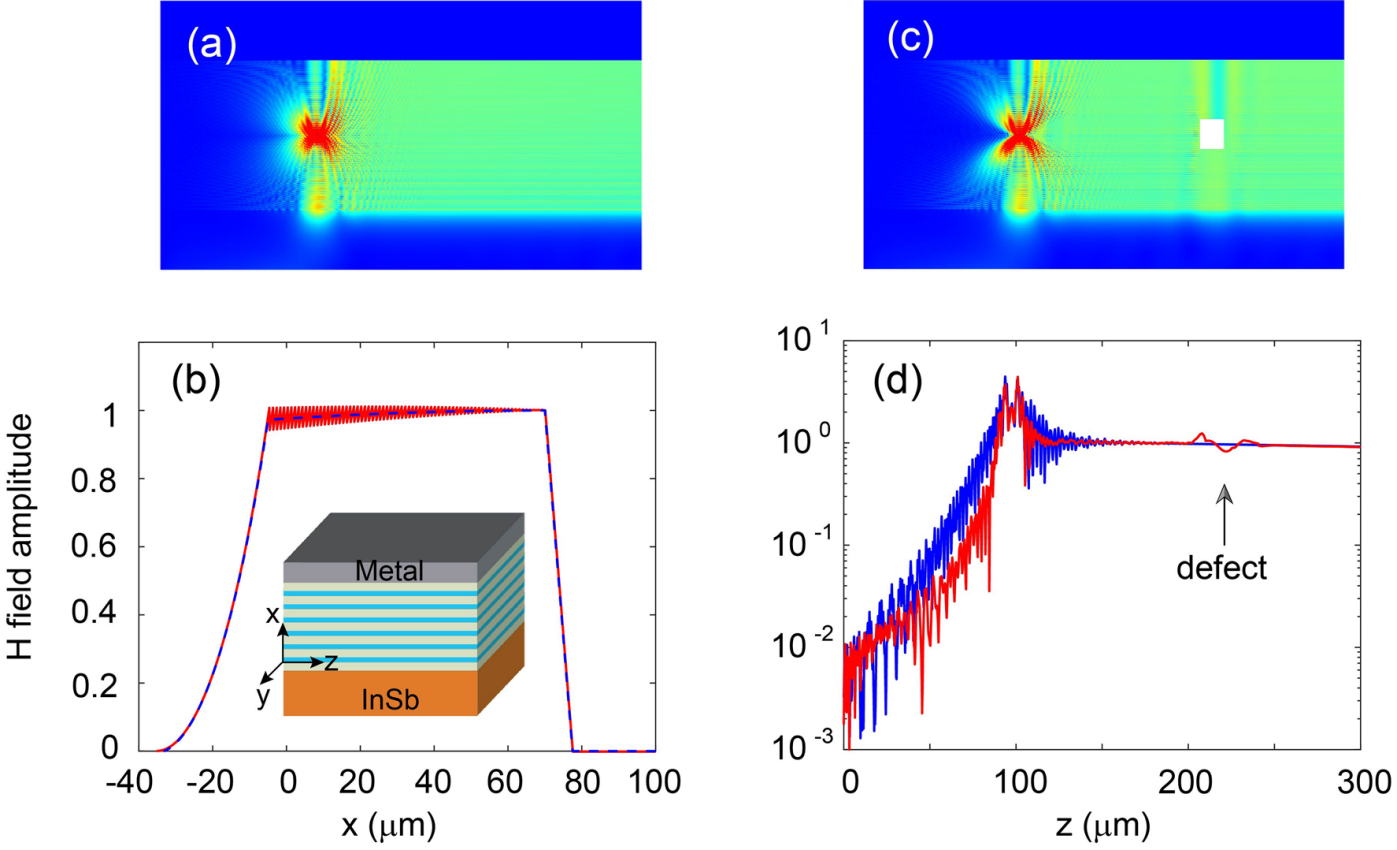
(**a**, **c**) Simulated H-field amplitudes in the realistic waveguide without and with defect. The UENZ layer in the waveguide consists of alternative plasmonic and Si layers, and the period is 1 $\upmu$m. The defect is a square Si column of side length 15 $\upmu$m. (**b**) H-field distribution along the *x* axis. The dashed line represents the corresponding H-field distribution for the effective model where the UENZ layer is homogenized. The inset is a schematic of the realistic waveguide structure. (**d**) Distributions of H-field amplitudes along the waveguide axis in (**a**) and (**c**). The frequency is 2.12 THz for all cases.

In this paper, the semiconductor is assumed to be *n*-doped InSb with $\varepsilon _\infty =15.68$ and $\omega _p=4\pi \times 10^{12}$ rad/s^20,21^, and we set $\omega _c=0.25\omega _p$, which corresponds an external magnetic field of 0.25 T. Figure 1b shows the dispersion diagram for the waveguide with $\varepsilon _z=0.001$, $\varepsilon _x=50$, $d_1=0.0042\lambda _p$ and $d_2=2\lambda _p$ (where $\lambda _p=150$ $\upmu$m, is the vacuum wavelength for the plasma frequency). There exist two pairs of SMP branches (red lines). The lower pair of SMP branches are located in the lower bandgap of the magnetized InSb, and they have different asymptotic frequencies as well as different upper cutoffs. In the frequency range from the upper cutoff of the right branch to the asymptotic frequency of the left branch, SMP is only allowed to propagate in the backward direction, and this is just a unidirectional frequency window in the lower bandgap. The upper pair of SMP branches have different lower cutoffs, thus creating another unidirectional frequency window in the upper bandgap. For our waveguide, USMP in the upper frequency window exists with small wavenumbers, and it is only allowed to propagate forward. The unidirectional frequency windows of two types are indicated by the yellow shaded areas in Fig. 1b. Here, it should be noted that if the Si layer is removed from our waveguide, the SMP branches in the lower bandgap will extend into the upper bandgap, leading to the disappearance of the upper unidirectional window. Besides, recent studies show that the asymptotic frequencies of SMPs vanish when the nonlocal effect of semiconductor is included, and consequently, the waveguide loses its lower unidirectional window^20,21^. This nonlocal response is a result of the free electron movement during an optical cycle, caused by convection and diffusion. The nonlocal effect can significantly modify the dispersion feature of SMPs, implying that the local model is inadequate. Therefore, in what follows, we focus on USMPs in the upper bandgap of the magnetized InSb. For our waveguide, it is also feasible that the unidirectional window covers the whole upper bandgap, as illustrated in Fig. 1d, where $\varepsilon _x=300$ and $d_1=0.0012\lambda _p$, and the other parameters are the same as in Fig. 1b.

In the nonlocal model, we adopt a hydrodynamic model of free-electron gas (without considering diffusion effects) to describe the semiconductor. From a microscopic hydrodynamic equation of motion for the free electrons that includes a pressure term, the free-electron current $\textbf{J}$ induced by the field in the semiconductor satisfies the following equation

5 $$\begin{aligned} {\beta ^2\bigtriangledown \left( \bigtriangledown \cdot {\textbf {J}} \right) +\omega \left( \omega +i\nu \right) {\textbf {J}}=i\omega \left( \omega _{p}^{2}\varepsilon _0\varepsilon _{\infty }{} {\textbf {E}}-{\textbf {J}}\times \omega _c \hat{x} \right) }, \end{aligned}$$

where $\beta$ is the nonlocal parameter. Here, we take $\beta = 1.07 \times 10^6$ m/s for InSb at room temperature^20^. The wave propagation in the magnetized semiconductor is governed by Maxwell’s equations in the form

6 $$\begin{aligned} \begin{array}{l} {\bigtriangledown \times {\textbf {E}}=i\omega \mu _0 {\textbf {H}}},\\ {\bigtriangledown \times {\textbf {H}}=-i\omega \varepsilon _0 {\textbf {E}}+{\textbf {J}}}. \end{array} \end{aligned}$$

By simultaneously solving the Maxwell’s equations combined with Eq. (5), the dispersion equation for TM-polarized bulk modes in the semiconductor can be derived as

7 $$\begin{aligned} \beta ^2 k_{b}^4 + [(1+\varepsilon _{\infty }\beta ^2)(\omega _{p}^2-\omega ^2)+ \omega _{c}^2]k_{b}^{2} +\varepsilon _{\infty }[(\omega ^2-\omega _{p}^2)^2-\omega _c^2\omega ^2]=0, \end{aligned}$$

where $k_b$ is the bulk-mode wavenumber. Compared to the local dispersion relation of bulk modes, $k_b=\pm \sqrt{\varepsilon _{\mathrm{{v}}}}k_0$, the nonlocal dispersion Eq. (7) implies that there exist two types of bulk modes in the nonlocal semiconductor. The green shaded areas in Fig. 1b depict the bulk-mode dispersion of InSb, which differs from that of the local model. In the nonlocal scenario, the lower bulk mode has no upper cutoff and covers the upper bandgap for the local model, while the upper bulk-mode zone remains similar to that for the local model and still exhibits a high-frequency bandgap.

**Figure 5:**
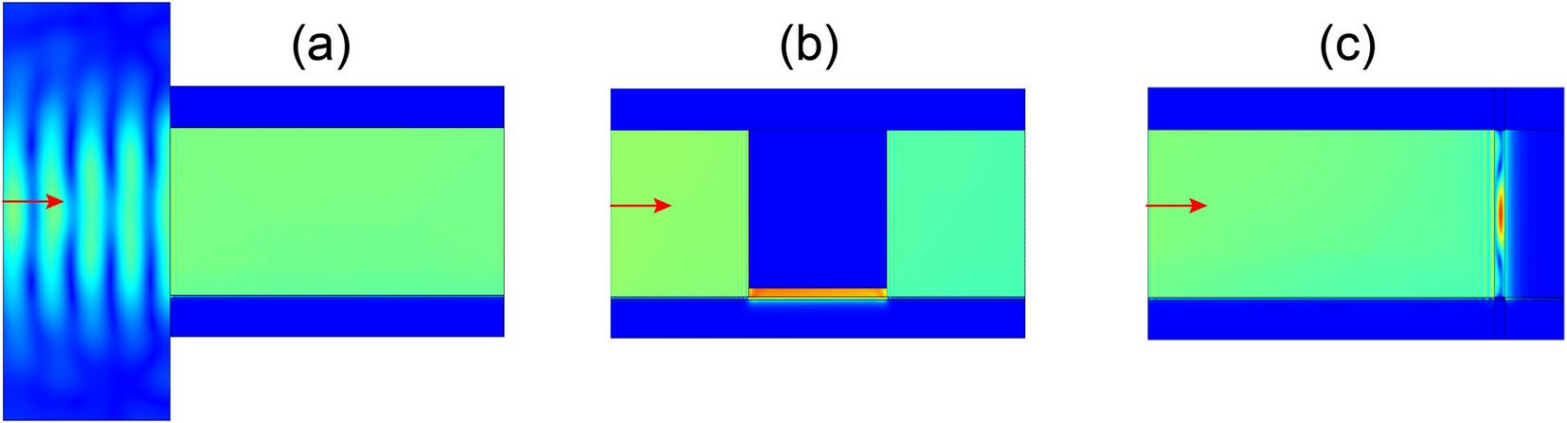
Simulated H-field amplitudes in systems where LUSMPs are coupled or manipulated at $f = 2.12$ THz. (**a**) LUSMP is efficiently coupled by an incident Gaussian beam with width $\lambda _p$. (**b**) Modal size of LUSMP is tailored by varying the UENZ-layer thickness. It is sharply reduced from $2\lambda _p$ to $0.2\lambda _p$, and then back to $2\lambda _p$. In the middle section, the modal energy is largely squeezed. (**c**) Complete trapping of LUSMP. The transverse size of the generated hot spot is equal to $\lambda _p$. The loss of the semiconductor is $\nu =0.002\omega _p$, and the other parameters are the same as in Fig. 1b.

Therefore, for the waveguide in the nonlocal model, the field of a SMP mode has two bulk-mode components in the semiconductor. In order to solve for the SMP modes in the waveguide with nonlocality, at the interface between the semiconductor and Si, an addition boundary condition of $J_x=0$ must be imposed, except for the ordinary boundary conditions, which require the continuity of the field components $H_y$ and $E_z$. As in the local case, the dispersion relation for the SMP modes in the nonlocal case is analytically derived, and it can be written as

8 $$\begin{aligned} \frac{(\sigma _2-\sigma _1)(k^2-\varepsilon _{\infty }k_{0}^{2})+k(\gamma _1-\gamma _2)}{\varepsilon _{\infty }(\gamma _1\sigma _2-\gamma _2\sigma _1)} + \frac{(\alpha _d/\varepsilon _d)\textrm{tanh}(\alpha _d d_1)+(\alpha _e/\varepsilon _z)\textrm{tanh}(\alpha _e d_2)}{1+(\alpha _e \varepsilon _d/\varepsilon _z \alpha _d) \textrm{tanh}(\alpha _d d_1) \textrm{tanh}(\alpha _e d_2)}=0, \end{aligned}$$

with

$$\begin{aligned} \begin{array}{l} {\sigma _1=\frac{\varepsilon _{\infty }k_{0}^2(\beta ^2 k^2 - \omega ^2 + \omega _{p}^2)-\gamma _1 \omega (\gamma _1\omega +k\omega _c)}{\varepsilon _{\infty }k_{0}^2(\beta ^2 k\gamma _1 + \omega \omega _c)-k \omega (\gamma _1\omega +k\omega _c)}},\\ {\sigma _2=\frac{\varepsilon _{\infty }k_{0}^2(\beta ^2 k^2 - \omega ^2 + \omega _{p}^2)-\gamma _2 \omega (\gamma _2\omega +k\omega _c)}{\varepsilon _{\infty }k_{0}^2(\beta ^2 k\gamma _2 + \omega \omega _c)-k \omega (\gamma _2\omega +k\omega _c)}}, \end{array} \end{aligned}$$

where $\gamma _1=\sqrt{k^2-k_{b1}^2}$ and $\gamma _2=\sqrt{k^2-k_{b2}^2}$ ($k_{b1}$ and $k_{b2}$ are the wavenumbers of the two bulk modes in the magnetized semiconductor). The dispersion Eq. (8) is numerically solved for the case in Fig. 1b, and the results are also plotted as dashed lines in this figure. In the nonlocal case, the asymptotic frequencies of SMPs in the lower bandgap of the magnetized InSb vanish, resulting in the disappearance of the USMP phenomenon. In the upper bandgap of the magnetized InSb, the dispersion curves of SMPs are identical for both the local and nonlocal cases, and this is more clearly seen in Fig. 1c. Besides, no backward mode emerges within the validity range ($\left| k \right| < 150k_p$) of the hydrodynamic model. So USMP and its unidirectional window in the upper bandgap are preserved in the presence of nonlocality. This is also the situation for the case in Fig. 1d. However, as the lower bulk mode becomes propagating in the (local) upper bandgap, this USMP become a leaky mode, because its field has a radiation component in the InSb. Consequently, the propagation constant *k* of USMP becomes a complex number in the nonlocal model. Therefore, it is necessary to further investigate the impact of the nonlocal effect on the modal properties of the USMP.

## Mode analysis and transmission simulation

In our waveguide, the USMP has a H-field distribution in the UENZ layer, characterized by $\alpha _e$ in Eqs. (3) and (8). As $\varepsilon _z$ close to zero, $\alpha _e$ approaches zero as well, allowing for a large modal area when $d_2$ is large. To demonstrate this, we calculated the H-field profiles of USMP at $f=2.12$ THz for both the local ($\beta =0$) and nonlocal $\beta = 1.07 \times 10^6$ m/s) cases, and the results are plotted in Fig. 2a, where $\varepsilon _z=0.001$, $\varepsilon _x=50$, $d_1=0.63$ $\upmu$m, and $d_2=300$ $\upmu$m. In the UENZ layer, the H-field extends uniformly in the both cases, resulting in a transverse modal size of USMP that can be represented by $d_2$. This modal size is nearly two (vacuum) wavelengths, implying that this USMP is a LUSMP mode. By using a UENZ material with $\varepsilon _z=0.001$ and $\varepsilon _x=50$, the maximal modal size of USMP can reach up to $14.1\lambda _p$. It should be noted that for the nonlocal model, the H-field amplitude does not decay to zero in the lossless InSb layer. As the propagation constant of LUSMP is smaller than the wavenumber of the additional bulk mode, the LUSMP mode has a radiation component in the semiconductor and thus becomes a leaky mode with a complex propagation constant $k=k_r+ik_i$, where $k_r$ is the phase constant and $k_i$ is the attenuation constant. It should be emphasized that due to the radiation loss, $k_i$ is nonzero even if the materials in the system are lossless. Our numerical calculations show that $k_i$ is closely dependent on $d_2$. Figure 2b depicts the dependence of $k_i$ on $d_2$ for the nonlocal ($\beta =1.07\times 10^6$ m/s) and lossless ($\nu =0$) case, where the frequency is $f=2.12$ THz, and the other parameters are the same as in Fig. 2a. $k_i$ decreases as $d_2$ increases. As $d_2$ increases from 0.1 to $2\lambda _p$, $k_i$ decreases from 0.04 to $0.0035k_p$. It is worth noting that at the same frequency, $k_i$ for the regular USMPs (RUSMPs)^21^ is approximately $0.04k_p$. Obviously, compared to RUSMPs, the radiation loss due to the nonlocality is largely reduced by an order of magnitude for the present LUSMP. For the LUSMP, the modal energy is primarily concentrated in the UENZ layer, effectively weakening the radiation effect in the semiconductor. This makes LUSMPs have a significant advantage over RUSMPs at terahertz frequencies.

**Figure 6:**
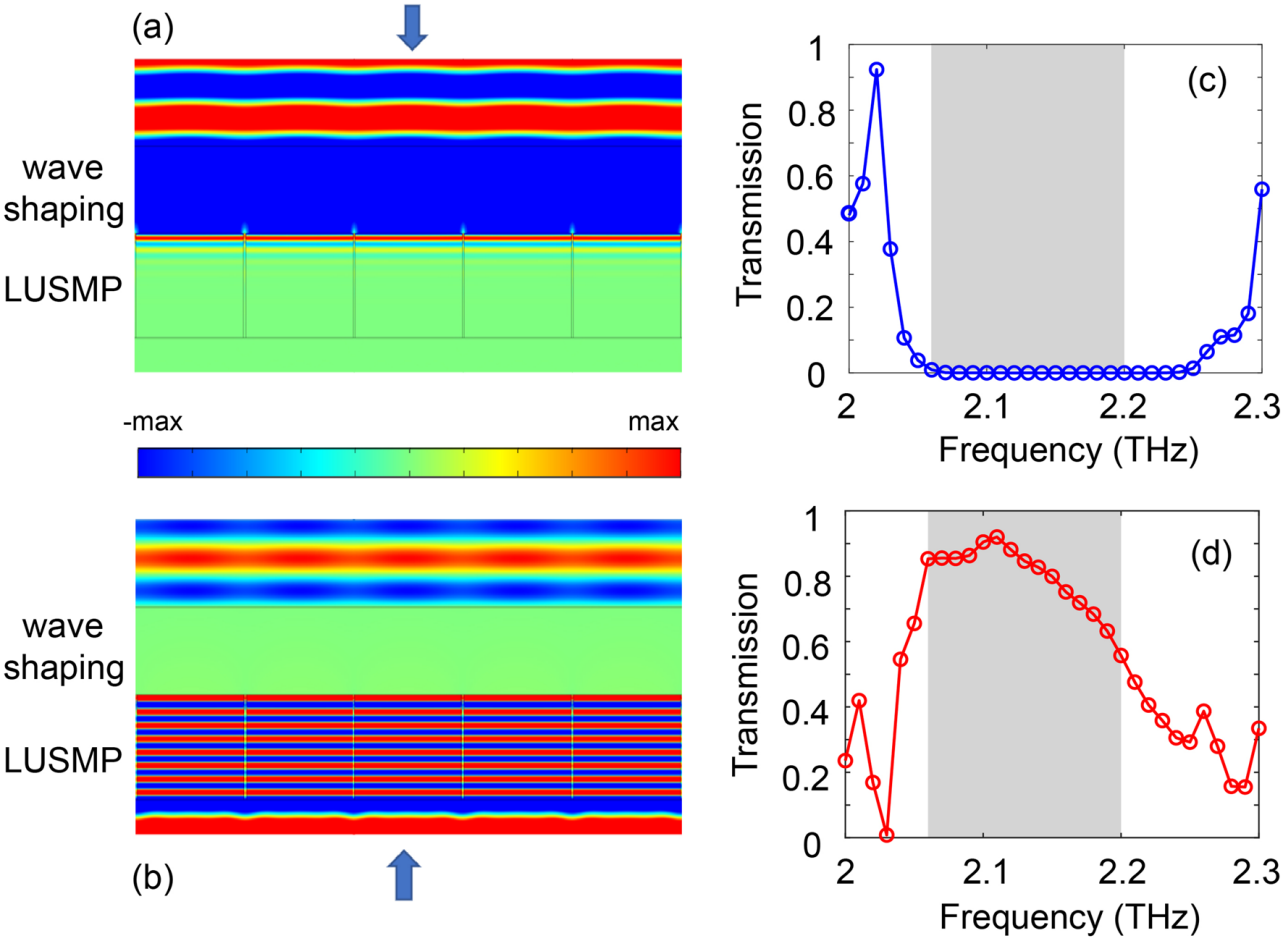
Terahertz free-space isolator formed by an array of LUSMP waveguides. (**a**, **b**) Simulated magnetic field amplitudes for plane waves incident from the top and bottom of the isolator. (**c**, **d**) Transmission efficiencies of the isolator for the forward and backward directions. The loss of the semiconductor is $\nu =0.002\omega _p$, and the parameters are the same as in Fig. 1b.

In the lossy case ($\nu \ne 0$), $k_i$ has two components: radiation loss due to nonlocal effect and absorption loss due to lossy materials. Figure 2b shows that the total $k_i$ of USMP in the nonlocal and lossy cases decreases as $d_2$ grows. Various values of the loss parameter $\nu$ are analyzed, including $\nu =0$. Evidently, the absorption loss of USMP can also be effectively reduced by increasing $d_2$, because of the same reason for decreasing the radiation loss. To further clarify this, we also present the dependence of $k_i$ on $d_2$ for the local cases with different $\nu$ values in Fig. 2c. In the local case, $k_i$ is only caused by the absorption loss, and it is indeed found to be decreasing with increasing $d_2$. Therefore, our guiding system can effectively reduce both radiation loss and absorption loss by increasing $d_2$. We further compare the propagation lengths [$L=1/(2k_i)$] of the LUSMP and the corresponding RUSMP in Ref. 21, which is the only example of true USMP previously reported. Figure 2d shows the propagation lengths of the LUSMP and RUSMP as functions of frequency. For our waveguide, the parameters are as follows: $d_1=0.63$ $\upmu$m, $d_2=300$ $\upmu$m; $\varepsilon _z=0.001$, $\varepsilon _x=50$. At $f=2.12$ THz, we find that for $\nu =0$, the propagation lengths are $L_{\mathrm{{RUSMP}}}=231$ $\upmu$m and $L_{\mathrm{{LUSMP}}}=3402$ $\upmu$m. When $\nu =0.002\omega _p$, the propagation lengths become $L_{\mathrm{{RUSMP}}}=166$ $\upmu$m and $L_{\mathrm{{LUSMP}}}=2883$ $\upmu$m, implying that the radiation loss is a dominating factor of the LUSMP and RUSMP losses for low-level material loss. However, when $\nu =0.02\omega _p$, we find that $L_{\mathrm{{RUSMP}}}=48$ $\upmu$m and $L_{\mathrm{{LUSMP}}}=1215$ $\upmu$m, which are close to their values of 77 $\upmu$m and 1650 $\upmu$m obtained from the local models, implying that the absorption loss dominates in this case.

To confirm the validity of LUSMP in the nonlocal model, we simulated wave transmission in the proposed waveguide ($\beta = 1.07 \times 10^6$ m/s, $d_2=2\lambda _p$) using the finite element method (FEM). The simulation employed a magnetic current line source as an excitation, specifically operating at 2.12 THz (i.e., marked by A in Fig. 1c), and the source was placed at the middle of the UENZ layer. A material loss of $\nu =0.002\omega _p$ was considered. The simulated H-field amplitudes are depicted in Fig. 3a. Notably, the excited wave propagates only in the forward direction, and the H-field is uniformly distributed in the UENZ layer away from the source. To examine the robustness of LUSMP in the nonlocal model, we inserted a defect into the waveguide, and re-simulated the guiding system. This defect (defect I), located in the middle of the UENZ layer, is an air column with a square cross-section of side length $0.5\lambda _p$. But no backward wave is generated by the defect, and the incident LUSMP completely bypasses it and continues to propagate forward, as illustrated in Fig. 3b. The defect only modifies the local field around it, and behind it the field quickly recovers. This is also clearly displayed in Fig. 3g, where the distributions of magnetic field amplitude along the middle line of the UENZ layer are plotted for both cases with and without defect. To investigate the influence of the defect size and location, we further move the defect down by $0.5\lambda _p$ (defect II), or double its size (defect III). The simulated results are displayed in Fig. 3c,e, respectively. In both the cases, the incident LUSMP bypasses the defect, but the field behind the defect is somewhat weakened, which is more clearly shown in Fig. 3g. Evidently, in these cases, the defect enhances local field near it in the semiconductor, which increases the absorption dissipation. Even so, the LUSMP mode still suppress backscattering from defect. It should be indicated that in the lossless cases, the LUSMP can completely circumvent the defects II and III in the waveguide, as illustrated in Fig. 3d, f, h. We also numerically analyzed defects of different shapes, such as air columns with circular or triangle cross-sections, and the observed phenomenon is similar to that for air square columns.

## Realistic waveguide using metamaterial

For the LUSMP waveguide, the UENZ material is a key component, whose relative permittivity is close to zero in the yz plane but larger than 10 in the x direction. Such a material does not exist in nature, however, it can be artificially realized using metamaterial technology. Here, as an example, the UENZ material is formed by alternating plasmonic and silicon layers, as depicted in the inset of Fig. 4b, and the period is much smaller than the wavelength. The plasmonic material may be a highly doped semiconductor, whose plasma frequency is significantly larger than that for the InSb. Thus, in the realistic waveguide, the UENZ layer comprises of seventy-five unit cells, and the period is 1 $\upmu$m. This waveguide can be fabricated with state-of-the-art technology: the thick Si film is first deposited on the substrate InSb, then thin plasmonic material and Si layers (of the metamaterial) are alternatively deposited, by using plasma-enhanced chemical vapor deposition (PEVCD) in a low-temperature environment^26^, and finally, a metal (e.g. Au) film is deposited on the top (thin) Si layer via conventional electron-beam evaporation^27^. According to the effective medium theory^28,29^, the UENZ metamaterial can be characterized by an effective (relative) permittivity tensor described by Eq. (1), and its elements have

9 $$\begin{aligned} \begin{array}{l} {\varepsilon _z=\delta \varepsilon _p+\left( 1-\delta \right) \varepsilon _q},\\ {\varepsilon _x=\frac{\varepsilon _p \varepsilon _q}{(1-\delta )\varepsilon _p +\delta \varepsilon _q } }, \end{array} \end{aligned}$$

where $\delta$ is the filling ratio of the plasmonic material, $\varepsilon _p$ and $\varepsilon _q$ are the relative permittivities of the plasmonic material and silicon, respectively. To attain the desired values of $\varepsilon _z$ and $\varepsilon _x$, we set $\delta =0.09$, $\varepsilon _p=-116.8$ and $\varepsilon _q=11.68$.

We utilize the FEM to simulate wave transmission in this realistic waveguide. The UENZ thickness is taken to be 75 $\upmu$m (we chose a relatively small thickness to reduce the demand for computational resources), and the local model is considered. A magnetic current line source is placed in the middle of the UENZ layer to excite LUSMP in the system. The operation frequency is set at 2.12 THz. The simulated H-field amplitudes are plotted in Fig. 4a. We also numerically solved for modes in the realistic waveguide along the *x*-axis using FEM, and the results are shown in Fig. 4b, where the H-field profile is almost uniformly throughout the designed UENZ layer. A comparison with the corresponding homogeneous UENZ layer case, represented by a dashed line in Fig. 4b, shows good agreement. Therefore, the LUSMP in the realistic waveguide possesses a transverse modal size of approximately 75 $\upmu$m. In order to confirm the robustness of LUSMP in the realistic waveguide, a square silicon column with a sidelength of 15 $\upmu$m is introduced as a defect and placed at a distance of 150 $\upmu$m away from the source. The results are presented in Fig. 4c. It can be observed that LUSMP well circumvents the defect, and this is more clearly illustrated in Fig. 4d, which shows the distributions of H-field amplitude along the middle line of the UENZ layer in Fig. 4a,c. This confirms that LUSMP in the realistic waveguide is a robust unidirectional mode.

## Applications of terahertz LUSMPs

We finally numerically demonstrate several advantages of LUSMPs over RUSMPs. Firstly, LUSMPs have a large modal area on the wavelength scale, enabling them to be efficiently excited by waves incident directly from air. To show this, we use a Gaussian beam with a width of $\lambda _p$ to excite LUSMP in a waveguide with $d_2=2\lambda _p$, and the simulated H-field amplitudes are illustrated in Fig. 5a. It is found that $70.1\%$ of the incident power is coupled to the LUSMP. In contrast, the RUSMP only has a coupling efficiency of $4\%$. Secondly, the modal size of LUSMP can be tailored by adjusting the UENZ thickness, as illustrated in Fig. 5b, where the modal size is abruptly varied from 2 to $0.2\lambda _p$, and then back to $2\lambda _p$. During the whole propagation process, the wave energy of LUSMP is first squeezed into a narrow channel and field amplitude is largely enhanced, then followed by almost full recovery with slight power loss due to the material loss ($\nu =0.002\omega _p$). Finally, LUSMP can be completely trapped with a hot spot of wavelength size. To achieve this, we have employed a backward-LUSMP waveguide to block the forward LUSMP, and simultaneously inserted a thin PMC slab with a thickness of $0.1\lambda _p$ between two InSb layers under opposite magnetic fields to prevent wave leakage. The empty space between the UENZ layers in the two waveguides is filled with a dielectric with a relative permittivity of 18. The simulated H-field amplitudes are displayed in Fig. 5c, revealing the presence of a hot spot with a transverse size of 150 $\upmu$m. The large size of the hot spot avoids the risk of device burning-out.

Based on their large modal sizes and unidirectional propagation nature, LUSMPs can be utilized to develop a terahertz free-space isolator, enabling the transmission of plane waves in only one direction. The free-space isolator is comprised of two parts, as illustrated in Fig. 6. The lower part is an array of LUSMP waveguides of length $2\lambda _p$, and the upper part is a dielectric layer of thickness $1.25\lambda _p$. This dielectric layer has a very low relative permittivity of 0.004, and it is used to shape the output wavefront from the waveguide array. In each LUSMP waveguide, the InSb layer has a finite thickness of $0.05\lambda _p$, which is far smaller than the UENZ-layer thickness of $2\lambda _p$. For the proposed isolator, its frequency window is determined by the frequency range of the LUSMP waveguides. In the frequency window of this isolator, plane waves are prohibited to transmit through it when they are normally incident from its top, as illustrated by numerically simulated magnetic field distribution in Fig. 6a. On the contrary, plane waves are allowed to transmit through the structure when they are normally incident from below, as illustrated by numerically simulated magnetic field distribution in Fig. 6b. Figure 6c,d show the transmission efficiencies of the isolator for backward (incidence from top) and forward (incidence from below) propagation directions. At the central frequency 2.12 THz of the operation window [indicated by the shaded areas in Fig. 6c,d), the efficiency of forward transmission is nearly $88\%$. This also implies that incident plane waves can efficiently excite LUSMPs in the waveguide array, owing to their flat field profile. For backward transmission, our numerical calculations show that the isolation ratio reaches up to 60 dB at 2.12 THz.

## Conclusion

In summary, we have conducted a comprehensive analysis of the guiding properties of a waveguide composed of metal, UENZ material, silicon, and magnetized InSb. It has been shown that in the upper bandgap of the magnetized InSb, the waveguide can support LUSMP mode, whose field is almost uniformly distributed over the thick UENZ layer. This LUSMP mode can possess a mode size larger than the wavelength. Moreover, the LUSMP mode is robust against the nonlocal effects of the materials, since the upper bandgap opened by the external magnetic field is topologically nontrivial. In comparison to (true) terahertz USMPs reported previously, which suffer from serious leakage loss caused by the nonlocality, the leakage loss of our LUSMP is largely reduced by an order of magnitude, because its modal energy is mainly distributed in the thick UENZ layer. Furthermore, we have numerically demonstrated the robustness of LUSMP against various defects. The robust LUSMP has also been confirmed in a realistic waveguide structure, where the UENZ material consists of alternative layers of plasmonic material and Si. In addition, we have highlighted several advantages of LUSMPs over conventional USMPs, such as efficient excitation by incident waves from space, energy squeezing with adjustable modal size, and wave trapping with hot spot on wavelength scale. By utilizing a LUSMP waveguide array, we have also designed a terahertz free-space isolator, whose isolation ratio is up to 60 dB. We expect that our work will stimulate much research interest on nonreciprocal surface plasmons in the terahertz regime.

## Data Availability

The datasets used and/or analysed during the current study available from the corresponding author on reasonable request.
